# Granulomatous Interstitial Nephritis Associated With Adalimumab Therapy in a Patient With Rheumatoid Arthritis: A Case Report

**DOI:** 10.7759/cureus.116129

**Published:** 2026-09-12

**Authors:** Naif Alghamdi

**Affiliations:** 1 Nephrology, Security Forces Hospital, Riyadh, SAU

**Keywords:** acute kidney injury, adalimumab, granulomatous interstitial nephritis, interstial fibrosis and tubular atrophy, rheumatoid arthritis

## Abstract

We report the case of a 67-year-old female who presented with a history of asymptomatic rising of serum creatinine after receiving adalimumab therapy for rheumatoid arthritis (RA). A renal biopsy revealed granulomatous interstitial nephritis (GIN). After other causes of acute kidney injury were excluded, adalimumab was held, and steroid therapy was initiated. His kidney function improved. Based on this case report, adalimumab could be a rare cause of impaired kidney function and should not be considered a completely safe treatment or disregarded as a potential cause of renal complications, such as GIN, as diagnosed in our case.

## Introduction

Acute interstitial nephritis (AIN) is an inflammatory kidney disorder involving the tubulointerstitial compartment, characterized histologically by interstitial inflammatory cell infiltration and edema [1]. A broad range of etiologies has been implicated, including medications such as antibiotics and non-steroidal anti-inflammatory drugs (NSAIDs), infectious diseases, and systemic autoimmune conditions. Granulomatous disorders represent another important group of causes [2]. Granulomatous interstitial nephritis (GIN) is an uncommon histopathological form of interstitial nephritis, identified in approximately 0.5%-0.9% of kidney biopsies [3].

Tumor necrosis factor-alpha (TNF-α) inhibitors have become established therapeutic agents for several immune-mediated inflammatory disorders. Adalimumab, a TNF-α inhibitor, is used in the management of rheumatoid arthritis (RA), psoriatic arthritis, ankylosing spondylitis, Crohn's disease, psoriasis, and juvenile idiopathic arthritis [4]. Although its systemic adverse effects are well recognized, renal complications associated with adalimumab appear to be uncommon.

We describe a patient with RA who developed progressive kidney dysfunction while receiving adalimumab and was subsequently diagnosed with GIN on kidney biopsy. Withdrawal of adalimumab combined with corticosteroid therapy was followed by improvement in kidney function. This case was previously presented orally at the 5th Glomerulonephritis Conference, held in Dubai from April 25 to 27, 2025.

## Case presentation

A 67-year-old Saudi female with a background of RA, dyslipidemia, and gouty arthritis was referred to the nephrology clinic after noticing a gradual increase in serum creatinine from a baseline of 60 μmol/L (0.68 mg/dL), documented six months before referral, to 150 μmol/L (1.70 mg/dL) at the time of referral. Her only medication was adalimumab 40 mg administered subcutaneously once weekly, which she had been receiving for 12 months. She had previously been taking methotrexate, then stopped due to gastrointestinal side effects one year ago. Not taking allopurinol for gouty arthritis for more than three years ago with recurrent attacks managed with steroids. Last gout attack more than one year before referral. She denied any history of taking NSAIDs or proton pump inhibitors (PPIs). She had no history of vomiting, abdominal pain, diarrhea, decreased urine output, hematuria, urine dribbling, difficulty passing urine, frothy urine, epistaxis, hemoptysis, shortness of breath, chest pain, or cough. She had no skin rashes, joint pain, nasal ulcers, recent contrast use, or recent infections, and had not used antibiotics, any other new drugs besides recently started adalimumab, or herbal medications. In addition, the patient had no history of generalized fatigue or fever.

On physical examination, the patient’s vital signs were normal. She was fully conscious and showed no abnormalities in abdominal, respiratory, skin, joint, ocular, or neurological examinations. Her laboratory test results showed the following values: serum creatinine, 150 µmol/L (1.7 mg/dL); urea, 12.5 mmol/L; HCO₃⁻, 23 mmol/L; and potassium, 5.1 mmol/L. Urine analysis revealed white blood cells 20-30/high-power field (HPF), 5-10/HPF red blood cells, with no protein or bacteria. The albumin-creatinine ratio was 10 mg/g. No abnormalities were observed in the complete blood count, including the eosinophil count, white blood cell count, and hemoglobin levels. The erythrocyte sedimentation rate and C-reactive protein level were within normal limits. ANA, C3/C4, anti-dsDNA, cANCA, pANCA, angiotensin-converting enzyme, QuantiFERON-TB test, and Brucella antibodies were negative. Ultrasound of the kidney shows normal kidney size and shape (Table 1). A renal biopsy was performed (Figure 1). The biopsy showed a tubulointerstitial compartment is extensively infiltrated by a mononuclear cell infiltrate with frequent foci of tubulitis. At least two large non-necrotizing granulomatous foci are seen surrounding interlobular arteries in one corticomedullary core. The granulomas consist of epithelioid cells with some palisades and Langhans and foreign body-type giant cells. The inflammatory infiltrate consists mainly of CD3-, CD20-, and CD68-positive cells and fewer plasma cells. Routine stains for fungi, bacteria, and acid-fast bacilli are negative. All immunostains are negative. The pathological diagnosis was GIN. After we excluded other common causes of GIN, such as sarcoidosis, antibiotic-induced GIN, infections, and autoimmune diseases, adalimumab was stopped, and oral prednisolone was started at 1 mg/kg/day (50 mg orally once daily) for four weeks, then tapered down over 12 weeks to 5 mg and then stopped. The last serum creatinine level after four months of discontinuation of adalimumab was stable at 111 µmol/L (1.26 mg/dL), representing partial but incomplete recovery of kidney function compared with her previous baseline of 60 µmol/L (0.68 mg/dL) (Figure 2).

**Table 1:** Laboratory and diagnostic evaluation at presentation.

Investigation	Result
Serum creatinine	150 μmol/L (1.70 mg/dL); baseline 60 μmol/L (0.68 mg/dL)
Potassium	5.1 mmol/L
Bicarbonate (HCO₃⁻)	23 mmol/L
Urinalysis	WBCs 20-30/HPF; RBCs 5-10/HPF; no protein or bacteria
Urine albumin-to-creatinine ratio	10 mg/g
ANA, anti-dsDNA, C3/C4, cANCA, pANCA	ANA, anti-dsDNA, cANCA, pANCA: negative; C3/C4: normal
Infectious workup (QuantiFERON-TB and Brucella antibodies)	Negative
Angiotensin-converting enzyme (ACE)	Within normal limits
Kidney ultrasonography	Normal kidney size and morphology

**Figure 1:**
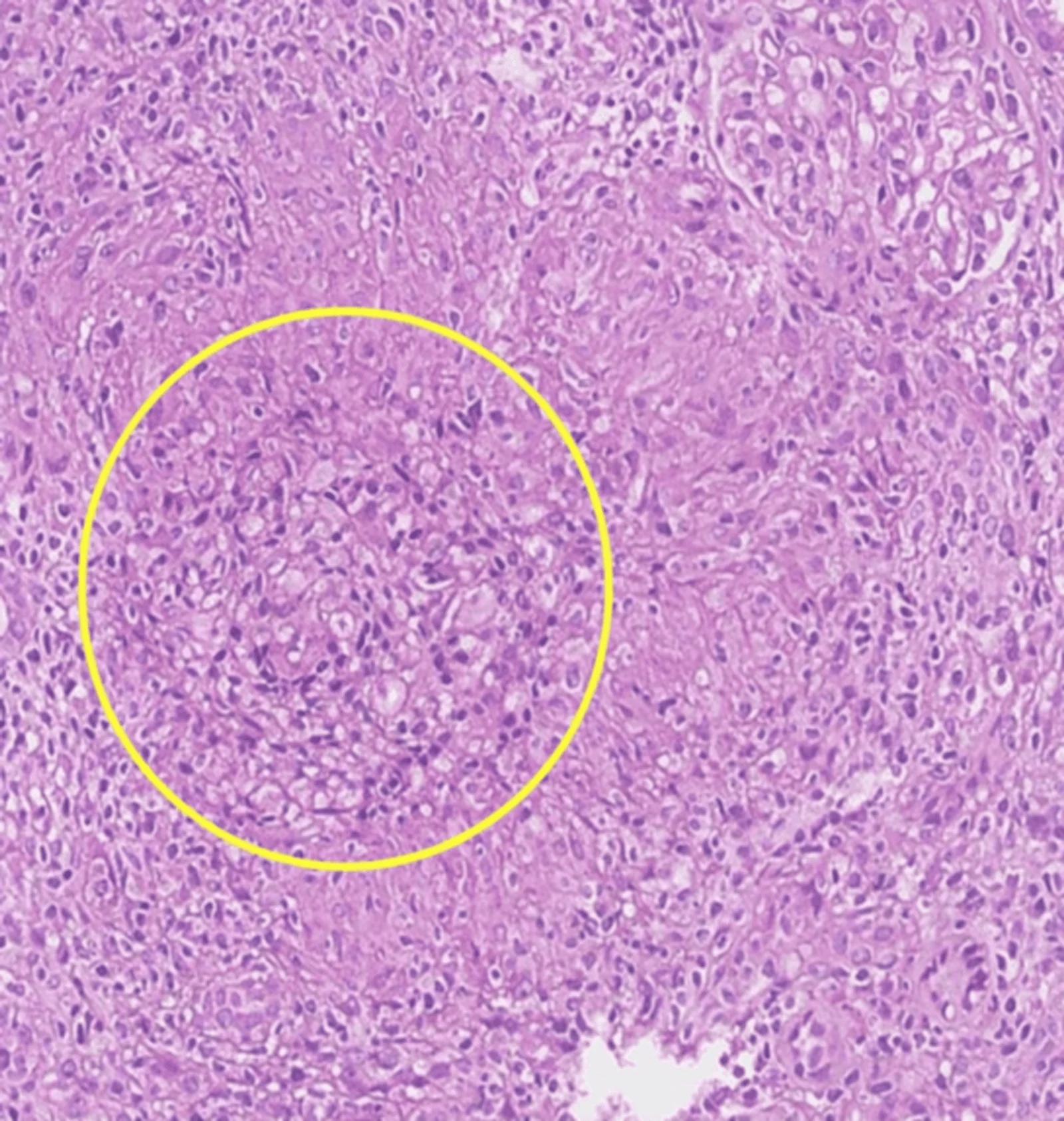
Light microscopic image of the renal biopsy specimen (hematoxylin-eosin (H&E) staining). The tubulointerstitial compartment is extensively infiltrated by mononuclear inflammatory cells with frequent foci of tubulitis. At least two large non-necrotizing epithelioid granulomas are present. The yellow circle highlights one of the non-necrotizing granulomas.

**Figure 2:**
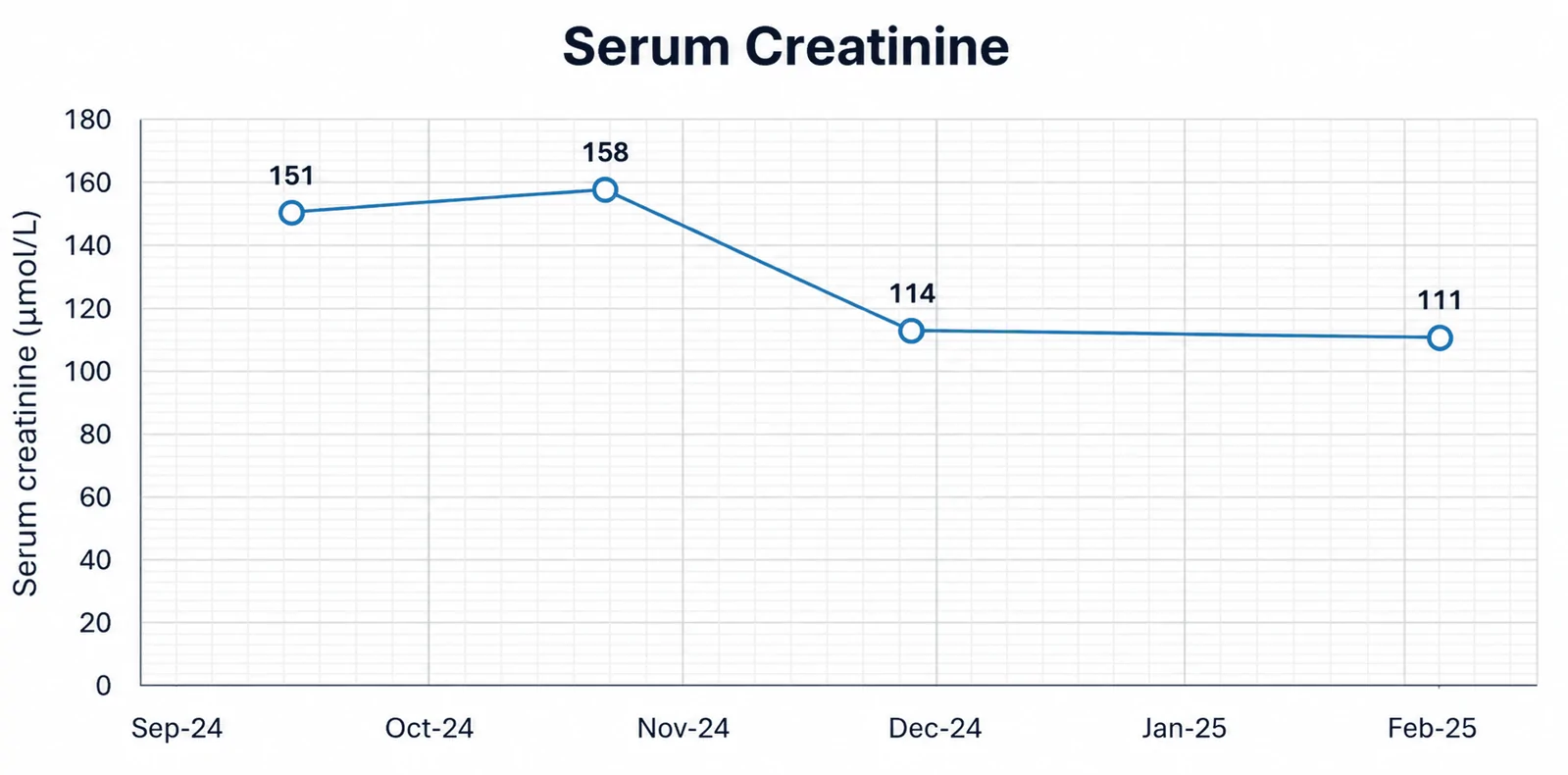
Serial serum creatinine measurements demonstrating partial recovery of kidney function during the clinical course.

## Discussion

RA can be associated with various renal manifestations, including glomerulonephritis, amyloidosis, and acute or chronic interstitial nephritis, while kidney injury may also result from prolonged NSAID use and, less commonly, biological therapies [5]. However, GIN is not a typical renal manifestation of RA. Furthermore, our patient had no evidence of active systemic or articular disease, normal inflammatory markers, and no significant proteinuria, making RA-associated renal disease less likely. GIN is an uncommon histopathological form of interstitial nephritis associated with conditions such as sarcoidosis, tuberculosis, fungal infections, and granulomatosis with polyangiitis, as well as medications including cephalosporins, vancomycin, nitrofurantoin, and ciprofloxacin [3]. Adalimumab-associated GIN is particularly uncommon, with only a few cases described in the literature [6-8], as summarized in Table 2. IgA nephropathy has also been reported as an uncommon renal complication associated with adalimumab [9]. Although the exact mechanism remains uncertain, TNF-α inhibition has been proposed to restore a T-helper type 1 (Th1) response with subsequent induction of interferon-γ, potentially promoting granuloma formation [10]. In our patient, adalimumab was considered the most likely cause of GIN and renal impairment after other recognized etiologies had been excluded. The interval between initiation of adalimumab and development of GIN varies, although available reports suggest that it may occur approximately 10-12 months after treatment initiation [5]. Withdrawal of the suspected causative agent, together with corticosteroid therapy, may help control renal inflammation and limit further kidney injury. Although non-necrotizing granulomas are less typical of tuberculosis, fungal infection, and granulomatosis with polyangiitis, stains for fungi and acid-fast bacilli were performed and were negative. Drug-induced disease and sarcoidosis remain important causes of non-necrotizing granulomatous inflammation. Although renal-limited sarcoidosis without extrarenal manifestations has been reported [11,12], the absence of extrarenal findings in our patient made sarcoidosis less likely. Other uncommon renal complications associated with adalimumab have been reported, including ANCA-associated vasculitis [13]. Renal recovery following GIN is variable and may remain incomplete, possibly because of residual fibrosis following resolution of granulomatous inflammation [14]. A notable feature of our case was the asymptomatic rise in serum creatinine without other prominent clinical manifestations, emphasizing the importance of considering kidney biopsy in patients who develop otherwise unexplained renal impairment while receiving adalimumab.

**Table 2:** Previously reported cases of adalimumab-associated granulomatous interstitial nephritis.

Author/year	Age/sex	Indication	Adalimumab duration	Creatinine (μmol/L)	Biopsy findings	Treatment	Outcome
Korsten et al., 2010 [8]	42/M	Ankylosing spondylitis	18 months	76 → 251.9	Interstitial mononuclear infiltrate with small, well-delineated noncaseating granulomata	Adalimumab discontinued + prednisone 0.5 mg/kg, subsequently tapered	Creatinine 127.3 μmol/L at 1 year; partial renal recovery
Sandys et al., 2018 [7] - Patient 1	42/M	Ankylosing spondylitis	20 months	82 → 168	GIN with non-caseating granulomas	Adalimumab discontinued	Creatinine stabilized at 120 μmol/L after 6 months
Sandys et al., 2018 [7] - Patient 2	58/M	Rheumatoid arthritis	5 years	97 → 250 → peak 555	Granulomatous nephritis without necrosis	Adalimumab discontinued + prednisolone 60 mg/day + hemodialysis	Dialysis-dependent for ~3 months; subsequently dialysis-independent; creatinine stabilized at 160 μmol/L
Plant et al., 2021 [6]	52/M	Psoriatic arthropathy	18 months	92 → 205	Chronic patchy tubulointerstitial nephritis with focal histiocytic aggregates forming loosely formed granulomata	Adalimumab discontinued; no corticosteroids	Progressive improvement; creatinine 110 μmol/L at 6 months
Alghamdi, 2026 (present case)	67/F	Rheumatoid arthritis	12 months	60 → 150	GIN with non-necrotizing epithelioid granulomas and tubulitis	Adalimumab discontinued + prednisolone	Creatinine improved to 111 μmol/L at 4 months; partial but incomplete recovery

## Conclusions

Adalimumab and other anti-TNF-α therapies are safe and very effective in managing different diseases, but renal function needs to be monitored regularly. Stopping the offending agent, with or without steroids, is the treatment strategy. Although kidney function may not fully recover to normal levels, the outcome is considered good.
